# Interactions between innate immunity and insulin signaling affect resistance to infection in insects

**DOI:** 10.3389/fimmu.2023.1276357

**Published:** 2023-10-17

**Authors:** Andrea M. Darby, Brian P. Lazzaro

**Affiliations:** ^1^ Department of Entomology, Cornell University, Ithaca, NY, United States; ^2^ Cornell Institute of Host-Microbe Interactions and Disease, Cornell University, Ithaca, NY, United States

**Keywords:** innate immunity, insulin signaling, metabolism, immunometabolism, humoral immunity, *Drosophila*, mosquito, *Bombyx*

## Abstract

An active immune response is energetically demanding and requires reallocation of nutrients to support resistance to and tolerance of infection. Insulin signaling is a critical global regulator of metabolism and whole-body homeostasis in response to nutrient availability and energetic needs, including those required for mobilization of energy in support of the immune system. In this review, we share findings that demonstrate interactions between innate immune activity and insulin signaling primarily in the insect model *Drosophila melanogaster* as well as other insects like *Bombyx mori* and *Anopheles* mosquitos. These studies indicate that insulin signaling and innate immune activation have reciprocal effects on each other, but that those effects vary depending on the type of pathogen, route of infection, and nutritional status of the host. Future research will be required to further understand the detailed mechanisms by which innate immunity and insulin signaling activity impact each other.

## Introduction

Innate immunity and insulin/insulin-like signaling (IIS) are both integral for homeostasis and anti-pathogen defense in insects. An immune response is critical for protecting an organism from invading pathogens that pirate resources and reproduce within the host (1, 2). The IIS pathway functions as a nutrient-sensing pathway that regulates cell and tissue growth, as well as whole-organism metabolism (3, 4). Innate immunity and IIS activity have been thought of as independent processes, but developments over the past decade have demonstrated them to be connected in mobilizing energy stores required for an effective immune response (5). In this review, we will discuss what we know of the physiological consequences and genetic mechanisms underlying interactions between insulin and immune pathways in insects, emphasizing primary examples from *Drosophila melanogaster* and integrating data from other insect models.

The innate immune system and IIS pathway are highly conserved across insect orders. Comparative genomics has revealed considerable conservation of orthologs in immune genes across almost all insects whose genomes have been sequenced (6). Similarly, insulin like-peptides (ILPs) that share structural and functional homology to mammalian insulin, have also been identified in major insect orders including Orthoptera, Diptera, Hymenoptera, Coleoptera, and Hemiptera (7–11). Concurrently, the development of genetic technologies from RNAi to CRISPR/Cas9 has enabled us to begin to understand how these pathways operate and how they impact each other across diverse insects (12–17), paving the way for future studies of how these systems interact to maintain organismal homeostasis in the presence and absence of infection.

## Effect of infection on metabolism and energetic stores

Mounting an immune response is an energetically costly process, requiring systemic physiological shifts in metabolism and energetic stores (16, 18–21). A broad range of pathogenic infections, from bacterial, viral, parasitic, or fungal, can lead to decreases in energetic stores like glycogen and triglycerides (12, 22–26). In insects, the fat body is a multifunctional tissue that can secrete antimicrobial peptides in response to immune stimulus and is also a site for the storage and breakdown of carbohydrates and lipids (27). Energetic stores like glycogen can be critical for supplying energy to activate the immune response. Glycogen is the polymeric form of glucose, and can be broken down in tissues like the fat body to be released into the hemolymph as free glucose by the enzyme glycogen phosphorylase (glyp) (28). *D. melanogaster* exhibit decreased glycogen levels and increased circulating glucose levels after infection with *Streptococcus pneumoniae*, with increased gene expression of *glyp* stimulated by the extracellular release of adenosine (25, 29). Phagocytic immune cells uptake the circulating glucose during the acute phase of *S. pneumoniae* infection, enabling phagocytosis*. D. melanogaster* deficient for the adenosine receptor or *glyp* exhibit lower levels of glycogen and higher mortality after *S. pneumoniae* infection (25, 29).

A metabolomics study on larvae of the silkworm *Bombyx mori* infected with the entomophathogenic fungi *Beauveria bassiana* found major shifts in hemolymph levels of metabolites like lipids, carbohydrates, and amino acids after infection (30). Fungal infection also caused higher levels of glucose and an associated reduction in trehalose (30). Trehalose is a disaccharide comprised of two glucose molecules that can be readily liberated into free glucose by the enzyme trehalase. Trehalose is the primary sugar found in insect hemolymph and is used for energetic needs like flight and metabolic homeostasis (28, 31). Although Xu et al. (30) did not measure trehalase activity, Praveena et al. (32) found that trehalase activity progressively diminishes in the hemolymph of *B. mori* larvae over five days of *B. beauveria* infection while they remain consistent in uninfected controls. These authors hypothesize that trehalase activity reduces over the course of the infection because the early hydrolysis of trehalose to glucose consumes the substrate pool available for conversion. The data suggest that during a fungal infection, trehalose may be converted to glucose to allocate energy to fighting the infection.

The breakdown of glycogen and trehalose provides free glucose for immune cell function (29). However, the role of triglyceride breakdown during infection is less well established. It has been recently demonstrated that *D. melanogaster* larvae exhibit reduced triglyceride stores during an active immune response, and shift from lipid storage to synthesizing phosphatidylcholine (PC) and phosphatidylethanolamine (PE) (16), which are two of the major phospholipids that comprise secretory vesicles and cell and organelle membranes (33). Active humoral immune responses drive high levels of AMP gene expression (e.g., 34), which could put a heavy burden for the endoplasmic reticulum (ER) to synthesize and secrete the encoded effectors into circulation. This shift to PE and PC synthesis during chronic immune activation has been hypothesized to support the secretion of immune effectors during the immune response.

Cellular stresses, including those imposed by immune reactions, stimulate the unfolded protein response (UPR) to allow the endoplasmic reticulum (ER) to regulate proper folding and secretion of proteins (35, 36). One marker of UPR in the ER is the upregulation of the transcription factor X-box binding protein 1 (XBP1), which positively regulates phospholipid synthesis and enzymes that increase the size of the ER (37). Both infection (34, 38) and chronic immune activation in the fat body (16) have been demonstrated to increase gene expression of *XBP1.* This elevated expression of ER-stress response genes is also associated with increased expansion of the ER lumen in infected mated females (38) and in uninfected larvae with an overactive immune response (16). These results suggest that the innate immune response induces ER stress, which increases phospholipid synthesis to support secretion of immune effectors. The infection-induced reduction of triglyceride stores observed across several *D. melanogaster* studies (12, 19, 23, 39) could arise from a need to mobilize energetic stores to support ER homeostasis by way of upregulating phospholipid synthesis.

**Figure 1:**
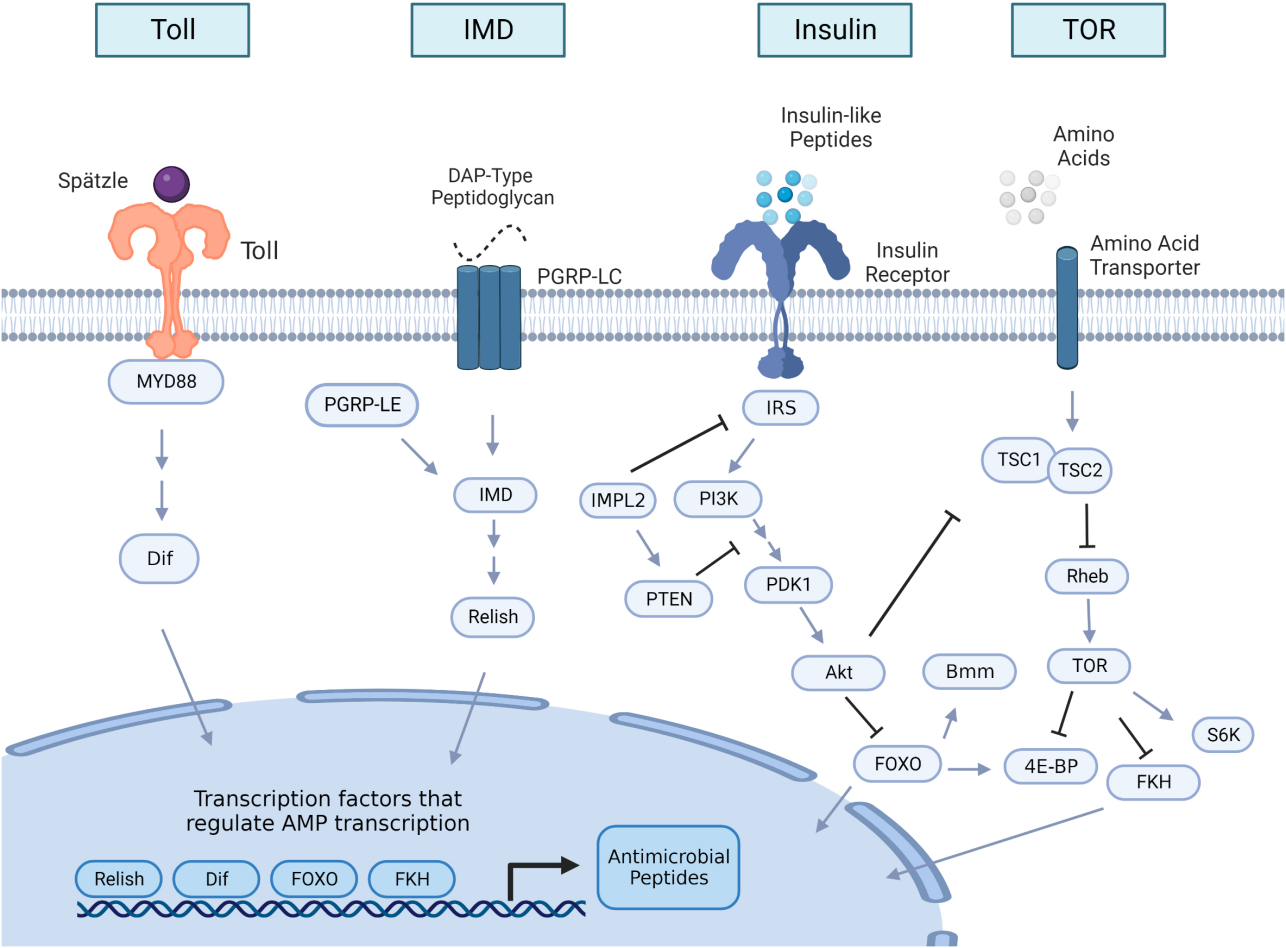
Insulin and innate immune pathway signaling overview in insects. The Toll and the immune deficiency (IMD) pathways are the two major innate immune signaling pathways that are induced upon bacterial or fungal infection, leading to the production of antimicrobial peptides (AMPs). The Toll receptor is activated by the cleaved form of the cytokine Spätzle, which is processed in a proteolytic cascade after the detection of Gram-positive bacteria or fungi. Once Toll is active, it initiates an intracellular cascade that leads to activation of the nuclear factor κB (NF-κB) transcription factor Dif. When activated, Dif translocate to the nucleus to initiate AMP transcription. IMD signaling is initiated by peptidoglycan recognition protein LC (PGRP-LC) or peptidoglycan recognition protein LE (PGRP-LE) binding DAP-type peptidoglycan shed by Gram-negative bacteria and some Gram-positive bacteria. This binding triggers a cascade that activates the NF-kB transcription factor Relish to translocate to the nucleus to transcribe AMPs. Insulin signaling activity begins with the production of insulin-like peptides (ILPs), which are predominantly secreted from insulin-producing cells and from other tissue sources like the fat body or muscle. An intracellular cascade occurs after ILPS bind to the insulin receptor, activating the protein Akt. Once Akt is active, it impacts multiple downstream processes within the cell, including inhibition of nuclear translocation of the forkhead family transcription factor, Forkhead box-O (FOXO), which can regulate transcription of AMPs, and the lipase brummer (bmm). Akt activation also stimulates the target of rapamycin (TOR) pathway by TSC1/TSC2 suppression. TOR is also activated by amino acid sensing. Downstream targets of TOR activation include repression of the forkhead box family member forkhead (FKH), which also regulates AMP transcription, activation of the protein kinase S6K, which is critical for growth processes, and the inhibition of translational regulator 4E-BP (eukaryotic initiation factor 4 binding protein). Arrows in the figure denote positive interactions and bars denote negative interactions. Double arrows indicate that additional steps in the pathway are not illustrated. IRS, insulin receptor substrate; IMPL2, ecdysone-inducible gene L2; PI3K; phosphatidylinositol 3-kinase; PTEN, phosphatidylinositol 3,4,5-trisphosphate 3-phosphatase; PDK1 (phosphoinositide-dependent protein kinase); TSC, tuberous sclerosis tumor suppressor Rheb, Ras homolog enriched in brain; S6K, ribosomal S6 kinase.

Sustained long-term infections can result in energetic wasting. In a healthy animal, insulin signaling regulates glucose homeostasis (40, 41). Phosphorylation of the IIS protein Akt leads to uptake of extracellular glucose (42) and promotes glycogen and triglyceride synthesis (43) by positively regulating the genes *glycogen synthase* and *acetyl coenzyme-A* (**Figure 1**; 44–46). Dionne et al. (12) demonstrated that *Drosophila melanogaster* infected with the bacterium *Mycobacterium marinum* exhibit impaired IIS activity over several days of infection. Reduction in phosphorylation of Akt leads to reduced expression of *glycogen synthase* and *acetyl coenzyme-A*. Consequently, flies infected with *M. marinum* become hyperglycemic and progressively lose glycogen and triglyceride stores until they ultimately die from the infection.

In examples such as this (12, 23) it can be difficult to determine whether the metabolic change reflects a manipulation of the host by the parasite for the parasite’s own nutritional benefit, or whether the wasting reflects a collateral cost of sustained immune reactions. In the case of bacteria in the genus *Mycobacterium*, the bacteria primarily depend on lipids like cholesterol as their source of carbon (47, 48) and a large portion of the genome for this genus is devoted to lipid metabolism (49). It is possible that wasting phenotypes observed in this infection could be a result of pathogen nutrient acquisition. This can be distinguished with appropriately designed experiments. For example, one human study investigated metabolic shifts induced by infection with *Mycobacterium tuberculosis* (Mtb), the causative agent of tuberculosis (TB). These authors measured metabolic shifts in blood samples from patients that were healthy, patients infected with Mtb and given short-term antibiotic treatment, and Mtb-infected patients without treatment (50). They found that Mtb infection, regardless of treatment, significantly shifted metabolic profiles compared to healthy patients, and they observed no significant differences in metabolites between TB patients treated with antibiotics or not, despite reduced pathogen load in patients given antibiotics (50). These authors suggest that their observation that antibiotic treatment does not impact infection-induced metabolic shifts implies that the host consumes metabolites during an active immune response. This study does not rule out the possibility that virulence factors produced by the pathogen could continue to impact host metabolism even after pathogen loads are reduced by antibiotic treatment. However, it can serve as an example of how to begin to study whether metabolic consequences of infection are due to host energetic reallocation or pathogen nutrient acquisition.

## Effects of dietary sugar on immune outcome

Both the immune system and insulin signaling are heavily influenced by dietary nutrition (51–55), thus any variation in the composition of diet can influence an organism’s ability to resist infection and regulate global metabolism. When *Drosophila melanogaster* are reared on high glucose (0.57 M), they experience reduced resistance to a systemic infection by the bacterial pathogen *Providencia rettgeri* (56). Similarly, those reared on high sucrose (1 M) experience increased mortality following infection by the pathogen *Pseudomonas aeruginosa* (52). High sucrose diets also impair immunological melanization in larval hemolymph (52) and reduce phagocytosis of fungal spores by *D. melanogaster* larvae (53).

IIS activity might mediate the higher post-infection mortality observed in flies fed high-sugar diets. Musselman et al. (52) found through an RNA-seq analysis that uninfected larvae with knockdown of the insulin receptor (InR) in the fat body exhibit elevated expression of AMPs while larvae with constitutive activation of InR in the fat body exhibit reduced AMP expression. Knockdown of InR in the adult fat body increases survivorship of systemic *P. aerugionsa* infection in flies fed a high-sugar (0.7 M) diet (52). These data suggest that reduction in insulin signaling could increase infection survival on high-sugar diets, potentially due to elevated expression of immune effectors. In that study, however, they did not specifically measure AMP expression during an infection in flies with InR knockdown so it remains to be determined whether InR knockdown promotes survival of infection by elevating AMP expression.

These systemic infections performed by Unckless et al. (56) and Musselman et al. (52) were performed on *Drosophila* reared on high dietary sugar throughout larval development. High-sugar diets cause developmental delays, lipidemia, hyperglycemia, and reduced body size in both larvae and adults (51, 52). Thus, the consequences of a high-sugar diet on adult defense could be due to dietary effects on larval development that may affect immune capacity in the adult stage. For example, *D. melanogaster* larvae fed high yeast diets exhibit increased expression of antimicrobials as adults (57). *Drosophila* larvae fed low-protein diets have lower counts of phagocytic immune cells (58) and are more susceptible to systemic *Pseudomonas entomophila* infection in the adult stage (59). Mosquito larvae fed low protein diets were more susceptible to *Plasmodium falciparum* (60) and Sindbis virus infections (61). Infection in the *D. melanogaster* larval stage can also affect adult fitness, including reduced lifespan (62) and smaller body size (63). Future research should distinguish between the immediate metabolic effects and developmental effects of sugar overnutrition. One strategy for doing this is to rear juveniles on the same rearing medium then transfer adults to varied experimental diets after development is complete. This approach could be directly compared to cohorts of insects that are reared on varied diets high in dietary sugar to determine the relative contributions of developmental history and immediate metabolic dysregulation on immune function.

Some studies suggest a potentially protective effect of dietary sugar during enteric infection. For example, *D. melanogaster* fed high-glucose diets (0.55 M) exhibit increased survival after an enteric *Vibrio cholerae* infection (64). Feeding sugar to the mosquito *Aedes aegypti* also increases expression of antiviral genes in the intestinal tract and protects mosquitos from oral arbovirus infections (65). High-sugar diets have been demonstrated to elevate reactive oxygen species levels (66) and increase expression of antimicrobial peptides (67) in the midgut of *D. melanogaster*. These data suggest high-sugar diets may alter gut immunity, potentially as a consequence of dysregulation of the endogenous microbiota (68), which would not occur in the hemocoel during a systemic infection. These data draw attention to important differences in the consequences of dietary sugar that may depend on whether the infection is acquired systemically or orally.

The dependency of infection-related phenotypes on dietary nutrition raises an important concern for comparisons across studies performed by different research groups. There is very little standardization of diets used for rearing *D. melanogaster* in infection studies (69, 70). For example, the diet used for the enteric infection referenced in (64) was a holidic (chemically-defined) diet, which is known to delay development relative to sugar-yeast based diets that are standard in *D. melanogaster* studies (71, 72), and the definition of “high” sugar varied nearly 2-fold between Musselman et al. (52) and Unckless et al. (56). Potentially, differences in dietary composition between studies could affect metabolism and infection phenotypes even when diet is not itself an explicit focus of the experiment.

## Consequences of innate immune activation on insulin signaling

Transcriptomic studies of infected insects often show differential expression of genes in gene ontology (GO) categories related to carbohydrate and lipid metabolism (34, 73–75). The IIS pathway is a critical regulator of carbohydrate and lipid metabolism (4, 76, 77). Thus, infection-induced shifts in carbohydrate and lipid metabolism could be mediated by alterations in IIS activity driven by the immune system.

The Toll signaling pathway (**Figure 1**) is one of two major innate immune signaling pathways in insects (78). Toll signaling is homologous to Toll-like signaling in vertebrates (79) and leads to activation of the NF-κB transcription factor Dif. Gram-positive bacterial and fungal pathogens primarily activate Toll signaling (80, 81). Activation of Toll in the *D. melanogaster* fat body either through genetic manipulation or infection suppresses insulin signaling as measured by reduced phosphorylation of Akt (19, 39, 82). Since the insulin pathway is critical for regulating growth and lipid metabolism (76, 77), inhibition of IIS activity by Toll activation during development results in reduced body size and depletion of triglyceride stores (19, 82).

Suzawa et al. (82) showed that infection of *D. melanogaster* larvae with the Gram-positive bacterium *Enterococcus faecalis* reduces production of insulin-like peptide 6 (dILP6), which is an ILP secreted from the fat body that regulates larval growth (83, 84). Overexpression of *Dif* in the larval fat body also reduces dILP6 mRNA transcripts, and the infection-induced reduction in dILP6 and growth inhibition can be rescued by RNAi knockdown of *Dif* (82). Roth et al. (39) further found that activation of Toll in the larval fat body inhibits PDK1 from phosphorylating Akt at residue T342, resulting in growth inhibition and reduced triglyceride storage (39). Body size and triglyceride storage were rescued by forced phosphorylation of Akt site T342 in the fat body of larvae with constitutively activated Toll (39). These results clearly place alteration of dILP6-production and larval insulin signaling downstream of the Toll signaling pathway. However, further experiments are required to test whether Dif directly regulates dILP6 expression or whether insulin signaling is secondarily regulated by proteins whose expression is directly controlled by Dif.

The immune deficiency (IMD) pathway (**Figure 1**) is the second main arm of the insect innate immune system (73). IMD signaling is responsive to DAP-type peptidoglycan shed by Gram-negative bacteria as well as some *Bacillus* species (81) and activates the NF-κB transcription factor Relish to induce transcription of antimicrobial peptides and other immune response genes (**Figure 1**; 81, 85). There is mixed evidence of IMD activity impacting insulin signaling and mobilization of energetic stores in *D. melanogaster*. DiAngelo et al. (19) found that constitutive activation of Relish in the fat body did not significantly impact Akt phosphorylation or triglyceride content (19). Although infection with the Gram-negative bacterium *Escherichia coli* reduced Akt phosphorylation, the suppression was lost when Toll pathway mutants were infected with *E. coli*, indicating that the attenuation of IIS was due to cross activation of the Toll pathway and not due to IMD signaling (19). In contrast, Davoodi et al. (86) found that constitutive activation of the IMD protein in the fat body significantly reduces phosphorylation of Akt and S6K throughout the body. S6K is a serine kinase downstream of Akt (**Figure 1**) that is critical for regulating larval growth (87). IMD activation in the fat body also reduced total triglyceride and trehalose levels in 3^rd^-instar larvae (86). Those larvae weighed less, had delayed development, and had reduced adult eclosion rates. These phenotypes are similar to those of insulin-signaling mutants and flies that overexpress Toll in the fat body (19, 88). One major difference between these two studies is the fly genotypes used to constitutively activate the IMD signaling pathway. Davoodi et al. (86) overexpressed the IMD protein where DiAngelo et al. (19) forced constitutive expression of the transcription factor Relish. The IMD molecule is an adaptor protein that acts upstream of Relish (**Figure 1**; 81), and, when activated, it triggers an intracellular signaling cascade that results in Relish activation (89). Potentially, there may be branching of the IMD pathway such that targets downstream of the IMD protein that disrupts insulin signaling and alter metabolic stores may not be regulated by Relish.

Davoodi et al. (86), also performed a transcriptomic analysis on larval fat body with constitutive IMD protein activation and identified modified expression of genes that regulate metabolic processes. Genes involved in the negative regulation of IIS were upregulated, including *impl2* and downstream targets of IIS like *4E-BP* (86). Lipid synthesis and gluconeogenesis genes were conversely downregulated in larvae with constitutive expression of the IMD pathway (86). These transcriptional data suggest that the IMD pathway, like Toll signaling, can influence metabolism.

Toll and IMD signaling are both critical for systemic immunity, however, the IMD pathway is primarily responsible for regulating gut immunity, including in response to endogenous microbiota (90). Impaired IMD signaling in the gut has been demonstrated to affect insulin signaling activity and metabolic stores. Kamareddine et al. (91) found a significant depletion of lipids in the fat body and an accumulation of lipid droplets in the anterior region of the midguts of several IMD mutants of *D. melanogaster* (*Dredd, key, RelE20, PGRP-LC and PGRP-LE*) in the absence of a systemic infection. Despite having depleted lipids in the fat body, whole-body triglyceride content and glucose levels were higher in the IMD mutants. These mutants also exhibited lower levels of *Ilp3* transcripts and reduced phosphorylated Akt in the intestine (91). Davoodi et al. (86) similarly observed that uninfected *imd* mutants have higher whole-body triglyceride levels and reduced expression of *Ilp2, Ilp3*, and *Ilp5*, although only in males. These results demonstrate that IMD signaling plays a critical role in regulating metabolic homeostasis even in the absence of systemic infection, potentially through actions in the gut or on the endogenous gut microbiota.

The insect digestive tract is a complex organ composed of multiple cell types that vary in function including immune defense, absorption of nutrients, and lipid metabolism (92). Enteroendocrine (EE) cells in the midgut epithelium produce hormones, regulate stem cell activity, and express insulin-like peptides (93). Kamareddine et al. (91) found that *Relish* knockdown in EE cells reduced *Ilp3* transcripts in the gut, increased EE lipid content, and reduced phosphorylation of Akt, while *Relish* knockdown in the fat body yielded no effect on Akt phosphorylation, triglyceride levels, or glucose content (91). Previous studies have demonstrated a role for commensal *D. melanogaster* gut microbiota like *Acetobactor pomorum* in regulating host metabolism, particularly via the microbial metabolic byproduct acetate (94, 95). Provision of acetate to germ-free flies increases nuclear localization of Relish and restores Akt phosphorylation (91). Germ-free wildtype flies have reduced IMD activity and exhibit metabolic phenotypes similar to those of flies with IMD knockdown in EE cells (91). Reciprocally, IIS activity and metabolic homeostasis are restored by overexpression of *Relish* in the EE cells of germ-free flies (91).

These data corroborate findings from an earlier microarray study that demonstrate microbiota-induced expression of host gene expression is *Relish*-dependent (96). Particularly, that study found that conventionally-reared flies exhibit upregulation of 285 genes in the gut relative to flies reared germ-free, and that GO categories for metabolic genes were enriched. When those authors then compared the transcriptomes of germ-free and conventionally reared Relish mutants, they found that expression of 151 of those 285 genes upregulated in response to microbiota was altered, including the insulin signaling genes *PI3K, InR*, and *thor* (96). Several other studies similarly demonstrate microbiota-Relish-dependent regulation of host gene expression in the gut. For example, oral infection with *Erwinia carotovora* also causes shifts in midgut gene expression that are *Relish*-dependent, including both immune and metabolic gene regulators (90). *Relish* mutants do not exhibit expression of microbiota-regulated genes observed in wildtype flies, regardless of whether they are germ-free or associated with microbiota (97). These data suggest a model whereby the host IMD pathway regulates the gut microbiota to maintain metabolic homeostasis, including through altered production of IIS activity by microbiota-derived metabolites such as acetate. However, the mechanistic details of signal integration under this model are unclear, and future experiments should study how downstream effectors of the IMD pathway interact with components of IIS to shape global metabolic regulation.

Since the IIS pathway is a critical global regulator of metabolism (76), it is also vital for regulating communication among tissues, and there are several studies that demonstrate innate immune activation also stimulating inter-organ communication in response to infection in *Drosophila* (98–100; see 101 for a review on inter-organ communication during an immune response). For example, glutamate derived from the muscle stimulates reallocation of lipids from the fat body to improve survivorship during oral infection with *Pseudomonas entomophila* (100). While not often directly investigated, there is evidence to suggest tissue communication during an immune response occurs in part by altered IIS activity. One study found that both systemic infection and overexpression of Toll and IMD in the fat body increased triglyceride and lipid droplet size in the midgut (99). This increase in lipid accumulation in the midgut was also associated with increased midgut gene expression of negative IIS regulators like *Impl2* and *thor* (99), which suggests that Toll and IMD activation in the fat body can suppress IIS activity in the midgut in response to innate immune activity in the fat body. In this study, lipid accumulation in the midgut was critical for surviving systemic *Photorhabdus luminsecens* infection (99). It is an exciting avenue of research to consider the extent to which innate immune activity and IIS activity may communicate across tissues to drive organism-level responses and increase survivorship during infection.

## Effects of insulin signaling on innate immunity

Several studies in *D. melanogaster* have characterized the effect that suppressing IIS activity has on infection outcome. The insulin receptor substrate *chico* is important for mediating the downstream cascade of IIS activity after insulin-like peptides bind to the insulin receptor (**Figure 1**, 76, 88). *Chico* mutants experienced increased survival of systemic infection by *Pseudomonas aeruginosa* and *Enterococcus faecalis* compared to wildtype flies (102). This higher rate of survivorship post-infection was not associated with increased levels of infection-induced gene expression of the AMP-encoding genes *Diptericin*, *Attacin*, and *Drosomycin*, although *chico* mutants do exhibit increased expression of *thor* in response to infection (102). Thor is an inhibitor of 5’-cap-dependent mRNA translation (103) and can prioritize translation of AMPs during an infection (104). Systemic infection with several different bacteria induces higher expression of *thor* (34, 104, 105) and *thor* mutants are highly susceptible to bacterial and fungal infection (104–106). Potentially, suppression of insulin signaling could be indirectly protective against infection due to downstream effects on cap-independent translation of proteins like AMPs.

A subsequent study conducted by McCormack et al. (107) found that *chico* mutants sustained lower pathogen loads after systemic infection with *E. coli* and *Photorhabdus luminescens*, although survivorship proportion was similar to wildtype flies. The effects on immune system activity were mixed, with reduced infection-induced transcription of the AMP genes *Diptericin*, *Cecropin A1*, and *Drosomycin*, and even reduced phagocytic capacity (107). However, the *chico* mutants exhibited increased phenoloxidase activity and melanization compared to wildtype. Melanization is important for insect wound healing and to clear pathogens at an injury site (108) and has been demonstrated to increase survivorship after bacterial and fungal infections (109). Potentially, increased phenoloxidase activity could also contribute to the increased survivorship of *E. faecalis* infection observed by Libert et al. (102) since melanization is also important for surviving *E. faecalis* infection (109). Similarly, higher phenoloxidase and melanization activity has been suggested to increase survivorship after *P. luminescens* infection (110, 111). However, melanization activity is not critical for surviving *E. coli* infection (112), thus improved phenoloxidase activity alone does not fully explain improved survivorship for *chico* mutants after *E. coli* infections observed by Libert et al. (102).

A more recent study assaying the effect of disrupted IIS activity on infection survival in *D. melanogaster* further demonstrated pathogen-specific infection outcomes. Davoodi et al. (86) delivered systemic and oral infections with five different bacterial pathogens (*Vibrio cholerae, Enterococcus faecalis, Pseudomonas sneebia, Providencia rettgeri*, and a virulent strain of *Serratia marcescens*) to triple mutants of *insulin-like peptides 2, 3*, and *5*.* P. sneebia* systemic infections resulted in 100% mortality for both wildtype and ILP mutants, while systemic infection for all other bacteria tested resulted in higher proportion of death in *ilp2,3,5* mutants. Pathogen loads were higher for *ilp2,3,5* mutants only after systemic infection with *P. rettgeri* and *E. faecalis.* In contrast, *ilp2,3,5* mutants showed higher survival of oral infection with *P. sneebia, V. cholerae* and *S. marcescens*, although only *V. cholerae* loads were reduced in *ilp2,3,5* mutants (86). When IIS activity is increased due to mutation in the IIS pathway antagonist *impl2*, flies suffered higher mortality and increased pathogen load than wildtype after oral *V. cholerae* infection (86). Collectively, these data suggest that functional IIS activity increases susceptibility to oral infections. *D. melanogaster* with oral infection of *V. cholerae* exhibit reduced activation of the IIS pathway (95), which further suggest that reduction in IIS activity could be a protective mechanism against enteric *V. cholerae* infections.

The combination of results across all studies demonstrates that the effects of IIS signaling on immunity varies with the specific pathogen and route of infection in *D. melanogaster*. Future experiments using tissue-specific approaches to manipulate IIS could elucidate how specific organs contribute to the interaction between IIS and immunity. Additionally, conditional genetic manipulations could be deployed to differentiate immediate immunological effects of altered insulin signaling from pleiotropic consequences of developmental effects due to constitutively altered IIS.

There is also evidence that IIS affects immunity in mosquitoes. For example, several studies demonstrate that *Plasmodium* infection results in IIS activation and host immune suppression in *Anopheles stephensi* mosquitoes (e.g., 113, 114)*. P. falciparum* infection induces the transcription of *insulin-like peptide 1, 3, 4*, and *5* in *A. stephensi* (10, 114). Artificial feeding of insulin-like peptide 3 (ILP3) and insulin-like peptide 4 (ILP4) to *A. stephensi* results in reduced expression of NF-kB-dependent genes in the midgut, including those encoding the antimicrobial peptides Gambicin, Cecropin, and Defensin (113, 115) and encapsulation-related proteins APL1, TEP1, LRIM1 (115). Similarly, feeding ILP4 or human insulin to *A. stephensi* at levels expected to be present in a bloodmeal (170 pM) induces IIS pathway activation and increases prevalence *Plasmodium falciparum* (113, 115, 116), where prevalence is defined as the proportion of mosquitos within the experimental population that become infected with *Plasmodium*. Reciprocally, inhibition of IIS activity with the PI3K inhibitor LY294002 or morpholinos that target *ILP3* and *ILP4* reduces the prevalence of *P. falciparum* infection and increases the expression level of immune effector genes in the mosquito midgut (113, 114). Together, these results demonstrate that insulin produced endogenously or obtained from a bloodmeal can suppress mosquito immune activity and could benefit infecting *Plasmodium.*

In a reciprocal experiment, Hauck et al. (117) demonstrated that overexpression of a negative regulator of Akt, phosphatase and tensin homolog (PTEN, **Figure 1**), in the *A. stephensi* midgut can reduce prevalence of *Plasmodium* infection. The proportion of PTEN-overexpressing mosquitos that became infected with *P. falciparum* was significantly lower than in wildtype controls and the transgenic mosquitoes developed fewer oocysts (117). Despite reduced prevalence of *Plasmodium* in the midgut, there was no effect on infection-induced expression of the AMP gene *Defensin* or in genes responsible for controlling *P. falciparum*, including *NOS, TEP1, APL1, LRIM1* (117). PTEN overexpression enhanced expression of genes that promote autophagy, which is associated with midgut integrity and improves infection resistance, and midguts that overexpressed PTEN were less permeable than midguts that overexpress Akt (117). Thus, enhanced midgut integrity may provide another mechanism for reduced prevalence of *Plasmodium* in IIS-suppressed mosquitoes.

Nevertheless, an independent set of studies suggested an opposite phenomenon. Corby-Harris et al. (13) generated transgenic *A. stephensi* lines that drive constitutive activation of Akt under the control of the midgut carboxypeptidase promoter to assay the effects that midgut Akt activation has on *Plasmodium* development. In contrast to the previously cited studies, they found that activation of Akt significantly reduced prevalence of oocysts in the midgut and the proportion of mosquitos infected with *P. falciparum* (13), with Akt overexpression impacting mitochondrial function and the production of nitric oxide species (NOS) (13, 118). NOS has been associated with killing *Plasmodium berghei* and *Plasmodium falciparum* (119–121). Suppression of NOS in mosquitos that overexpress Akt increases their susceptibility to *P. falciparum* infection, demonstrating that the Akt-induced suppression of *Plasmodium* prevalence is mediated at least in part by elevated NOS levels (122). Overexpression of Akt in the midgut also results in abnormal mitochondria morphology and significantly reduced number of mitochondria in the midgut (122), with mitochondrial dysfunction evident in reduced activity of Mitochondrial Complexes I, II-II, and V. Additionally, Akt-overexpression in the fat body of mosquitos systemically infected with *E. coli* or *Bacillus subtilis* led to higher induction of antimicrobial peptides and higher survivorship of infection (118). These findings suggest that activation of IIS pathway in the fat body and midgut can induce an immune response to limit *Plasmodium falciparum* development and control bacterial infections.

These inconsistency between studies showing that insulin suppresses the immune response and increases *P. falciparum* prevalence (113, 115–117) versus those showing that overexpression of Akt increases immunity and suppresses *Plasmodium* establishment (13, 118) may result from differences in IIS activity or expression pattern. Surachetpong et al. (116) demonstrated that feeding human insulin to *A. stephensi* increased ROS production and NOS synthesis but that the elevated NOS synthesis was not associated with reduced *P. falciparum* prevalence (116). Potentially, transgenic mosquitos that overexpress Akt in the midgut or fat body may synthesize higher levels of NOS and ROS than is produced in wildtype mosquitos fed insulin. This could be tested in future studies. Additionally, insulin may have other immune-suppressive effects on mosquito physiology independent of Akt activation. More refined experimentation, including tissue-specific transcriptomic contrasts between mosquitoes fed on insulin compared to those overexpressing Akt could help elucidate the cause of the differences between the studies.

Interestingly, insulin treatment of vertebrate macrophages similarly suppresses inflammation and innate immunity regulated by NF-κB transcription factors (123–126). For example, mouse macrophages primed with insulin prior to immune challenge with lipopolysaccharide (LPS) exhibited attenuated activity of Toll-like Receptor 4, significantly reduced nuclear localization of NF-κB, and reduced gene expression of Tumor Necrosis Factor alpha (TNFα) (126). When insulin-primed cells were treated with a PI3K inhibitor, insulin-mediated suppression of immune activity was alleviated (126). These data further suggest that insulin-mediated suppression of NF-κB activity is evolutionary conserved between vertebrates and invertebrates.

## TOR regulation of immune activity

Target of Rapamycin (TOR) is a nutrient sensing pathway that is jointly regulated by IIS activity and the abundance of free amino acids (**Figure 1**; 76, 127, 128). The Tsc1/Tsc2 complex negatively regulates TOR, and is inhibited by Akt (76). Thus, Akt phosphorylation activates the TOR complex to induce downstream cellular processes that promote growth (76). Inhibition of TOR can potentiate immunity in both mosquitos and Drosophila (129–131). For example, inhibition of *A. stephensi* TOR with rapamycin increased expression of genes like those encoding the NF-kB transcription factor *Rel2*, antimicrobial peptides *Attacin* and *Cecropin*, the complement-like protein thioester-containing protein 1 (TEP1), and the CLIP domain serine protease SPCLIP1 (130). TOR inhibition also reduced infection prevalence of *Plasmodium berghei* in *A. stephensi* (130). Similarly, suppressing TOR using rapamycin treatment in *D. melanogaster* increased expression of the antimicrobial peptides *Diptericin* and *Metchnikowin* (129), and also improved survival of systemic infection against the Gram-negative bacterium *Pseudomonas aeruginosa* (132).

Suppression of TOR through genetic manipulation has similar effects on immune function as rapamycin treatment (129, 132). For example, both uninfected *TOR* mutants and ubiquitous overexpression of the negative TOR regulator *Tsc1/Tsc2* increased *Diptericin* and *Metchnikowin* expression in *D. melanogaster* under conditions of high protein availability (129). RagA is a GTPase that positively stimulates TOR activity (133), and flies that expressed a dominant negative form of RagA (which suppresses TOR activity) exhibited increased survivorship after *P. aeruginosa* infection (132). Rheb is a GTP-binding protein that positively regulates TOR activity (134, 135). Overexpression of Rheb reduced expression of the AMP genes *Diptericin* and *Metchnikowin* (129). Interestingly, genetic suppression of TOR by overexpression of *Tsc1/Tsc2* increased susceptibility to systemic infection with the human pathogen *Burkholderia cepacia*, with flies exhibiting higher mortality and pathogen burden (136). TOR associates with two complexes, TOR complex 1 (TORC1) which is responsive to rapamycin and Tsc1/Tsc2 activity, and the less-studied TOR complex 2 (TORC2), which does not interact with Tsc1/Tsc2 or rapamycin treatment (137). When TORC2 is genetically suppressed via mutations lacking TORC2-specific components SIN1 and Rictor, flies exhibit increased survivorship and lower pathogen loads after *B. cepacia* (136). It is not clear to what extent TORC2 activity effects survivorship of other pathogens or its effects on immune capacity such as AMP expression.

TOR activity has also been recently demonstrated to play a role in *D. melanogaster* survival after oral infection. Deshpande et al. (131) discovered that active TOR signaling is necessary to survive an enteric infection with the entomopathogenic bacteria *Pseudomonas entomophila.* Oral infection with *P. entomophila* increases TOR activity and elevates expression of lipid synthesis genes like *fatty acid synthase 1* and *Lipin.* Suppression of TOR via rapamycin treatment decreases infection survivorship without affecting bacterial load or AMP gene transcription (131). The higher mortality post-infection observed in flies with suppressed TOR activity was associated with significant depletion of lipid stores, specifically in the fat body and anterior region of the gut. This reduction in lipid after infection is associated with decrease in expression of lipid synthesis genes and an excessive loss in lipid stores (131), and suggests that TOR can regulate lipid homeostasis for tolerance of enteric infection.

## Interactions between FOXO and active immune response

Forkhead box proteins are a family of transcription factors whose activity is suppressed by IIS (**Figure 1**) (138, 139). The forkhead box-O (FOXO) protein in particular is involved in various physiological functions including apoptosis, stress responses and metabolic homeostasis (140–142), which are conserved from invertebrates to vertebrates (139, 143–146). Activated Akt phosphorylates FOXO, preventing FOXO from localizing to the nucleus to drive transcription of target genes including those that mobilize metabolic stores (76). FOXO is responsive to varied stress stimuli including starvation, diapause, hypoxia, and infection (12, 147–151) and regulates processes like lipolysis and autophagy in response to these stressors (149, 152).

Even in the absence of infection, starvation induces FOXO-dependent expression of antimicrobial peptides in the gut, trachea, fat body, and epidermis of *D. melanogaster* that is independent of Toll and IMD signaling (153). Similarly, the transcription factor Forkhead (FKH) drives AMP transcription in *Drosophila* in the absence of infection as a function of inhibited TOR signaling, again independent of Toll and IMD signaling (129). Starving larvae of the silkworm *Bombyx mori* larvae also exhibit decreased Akt phosphorylation and elevated expression of the AMP genes *CecropinB6*, *Attacin1* and *Defensin B* (154). AMP gene promoters in varied insects frequently contain binding sites for forkhead-family transcription factors (153–156). These may allow infection-independent FKH/FOXO-regulated expression of these genes, and there may also be synergism between forkhead family and NF-kB family transcription factors. Future study should emphasize the extent to which forkhead-family transcription factors synergize with canonical immune signaling during an active infection.

Inhibition of FOXO results in increased nutrient storage and activation of FOXO results in nutrient mobilization and release. As could then be expected, *D. melanogaster* mutants for FOXO experience reduced loss of glycogen and a partial rescue of metabolic wasting compared to wildtype flies during a systemic *Mycobacterium marinum* infection (12). FOXO-deficient flies have increased survival of infection with *M. marinum* despite having pathogen load similar to that of wildtype flies, which suggests that the progressive loss of nutrient stores associated with activated FOXO increases mortality.

FOXO additionally has also been implicated in contributing to IMD-mediated effects on lipid metabolism in the absence of infection. Molaei et al. (149) found Relish mutants to have lower whole-body levels of triglyceride. Knockdown of Relish in the fat body had no effect on triglyceride content under normal rearing conditions, but when knockdown flies were fasted, they exhibited a significant reduction in triglyceride levels that was not seen in starved, wildtype flies (149). This drastic reduction in triglyceride levels in a Relish knockdown background was also associated with elevated expression of the lipase gene *brummer* (*bmm*). These authors determined that the depletion of lipids in starved Relish-deficient flies was due to FOXO-dependent regulation of *bmm*.

FOXO activates transcription of the lipase *bmm* (**Figure 1**) to positively regulate lipolysis (157, 158). Reducing FOXO activity in Relish mutants restores wildtype levels of fasting-dependent *bmm* gene expression and triglyceride storage in the fat body (149). In flies that are starved or fully fed, Relish can bind to the Bmm locus (149). Relish has been suggested to modify histone acetylation during starvation conditions demonstrated by enrichment of histone 3 lysine 9 acetylation (H3K9ac) in *relish* mutants at the site where Relish binds the Bmm locus (149) Together, these data suggest that Relish may antagonize FOXO-dependent regulation of lipolysis to prevent rapid loss of lipids during starvation. Future experiments could elucidate whether Relish-FOXO antagonism mediates the shifts in lipid metabolism observed during infection (12, 23, 131).

FOXO also plays tissue-specific roles during oral infections. During oral infection of *D. melanogaster* with *Serratia marcescens*, FOXO becomes activated and localized to the nucleus in gut epithelial cells, where it contributes to infection-induced expression of antimicrobial peptides (155). Even in the absence of infection, overexpression of FOXO in *D. melanogaster* intestines increases transcript levels of antimicrobial peptide genes *Attacin*, *Drosomycin*, *Diptericin*, and *Defensin* in gut cells (155). FOXO mutants experience reduced survivorship and higher bacteria load in response to oral infection with *S. marcescens* compared to wildtype, presumably as a consequence of the impaired AMP expression (155).

Recent evidence in *B. mori* demonstrates a role for FOXO in controlling infection by the orally-acquired baculovirus *Bombyx mori* nuclear polyhedrosis virus (BmNPV). *B. mori* cell lines infected with BmNPV exhibit increased phosphorylation of Akt (159, 160) and reduced FOXO gene expression (17). Phosphoenolpyruvate carboxykinase (PEPCK) is a downstream target of FOXO that is known for its role in regulating gluconeogenesis (161), and more recently for its antiviral role in *B. mori* (17, 162). *B. mori* cells overexpressing PEPCK exhibited a reduction in replication of BmNPV and elevated expression of the autophagy-related protein ATG8 (162). PGRP2-2 negatively regulates PTEN, which is itself a negative regulator of IIS (160). Thus PGRP2-2 is a positive regulator of IIS, and consequently a negative regulator of FOXO and PEPCK. *PGRP2-2* knockdown and *FOXO* overexpression reduce replication of BmNPV in a *B. mori* cell line (160) and increase expression of *PEPCK* and autophagy-related genes like *ATG6*, *ATG7*, and *ATG8* (17). Pharmaceutical inhibition of Akt phosphorylation in *B. mori* cells using a PI3K inhibitor also suppresses viral replication (160). These results suggest that FOXO plays a critical role in this antiviral immune response, and viruses like BmNPV can impact expression of its host IIS to evade the immune response.

Evidence from *D. melanogaster* also suggests FOXO mediates anti-viral immunity. Constitutive activation of FOXO can reduce the viral load of the Cricket Paralysis virus in *D. melanogaster* S2 cells (148). FOXO mutants are also more susceptible to RNA virus infections (148). FOXO regulates stress responses like autophagy and apoptosis that can be critical for combatting viral infection, providing a potential mechanism for FOXO-mediated resistance to viruses. The anti-viral effects of FOXO have thus far been predominantly evaluated in cell culture. Future experiments may determine whether FOXO is involved in tissue-specific antiviral responses and how those may impact host survivorship *in vivo*.

## Future directions and considerations in experimental design

**Figure 2:**
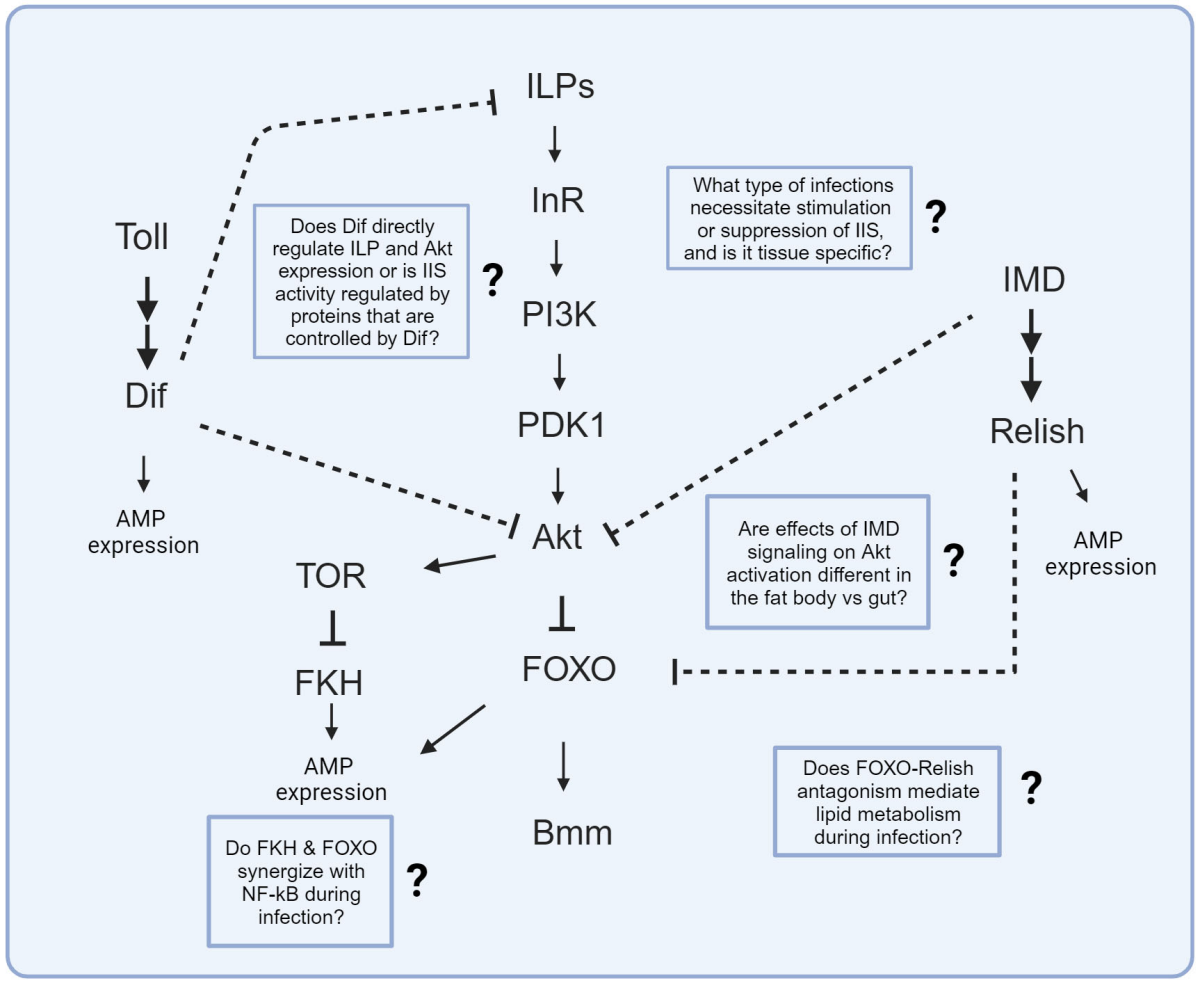
Known signaling interactions between innate immune and IIS/TOR pathways. Arrows depict known activation while bars represent known suppression in molecular interactions. Dashed bars represent known suppression between innate immune and IIS pathways, but the specific molecular interaction is unknown. Text boxes with question marks indicate outstanding questions regarding these signaling interactions that could be the study of future research.

In this review, we have discussed examples of known interactions between innate immune and IIS pathways in the model insect *D. melanogaster* as well as in insects of economic and public health relevance like *B. mori* and *A. stephensi*. **Figure 2** summarizes known interactions between Toll and IMD and IIS/TOR and questions to consider for further investigation. **Supplementary Table 1** contains a list of published experiments in *D. melanogaster, A. stephensi*, and *B. mori* that have evaluated how different pathogens and routes of infection (i.e., oral vs. systemic) impact IIS/TOR activity and immunological phenotypes. **Supplementary Table 2** summarizes several published studies in *D. melanogaster* that tested how manipulating the expression of innate immune genes affects IIS activity and IIS-dependent metabolism. The literature reviewed demonstrates a key role of insulin signaling in responding to oral, parasitic, viral, and systemic infections (12, 17, 19, 39, 115, 155). It also reveals critical differences between tissues, pathogens, life stages, and routes of infection in how insulin-immunity interactions play out. Different adaptive strategies of IIS regulation could be deployed by the host depending on the type of pathogenic infection. IIS and TOR upregulation could promote tolerance of pathogens that stimulate wasting of nutrient stores. For example, Akt and TOR are activated by oral infection with *P. entomophila*, which promotes lipid synthesis and alleviates infection-induced wasting to increase survivorship (131). But for infections that may not pose a threat of host wasting, suppression of IIS and promotion of FOXO-dependent signaling to regulate lipid metabolism and transcription of immune effectors may be more advantageous. Akt activation is inhibited after oral infection with *S. marcescens*, which activates FOXO to potentially help upregulate transcription of AMPs that may act as a defense at the gut epithelium (155). Potentially, FOXO-dependent regulation of AMPs in the gut epithelial barrier may enable a localized response that is more efficient than dumping antimicrobials into the gut lumen, where they would be at lower effective concentrations and could cause dysbiosis of the resident microbiota. Additionally, pathogens and parasites may manipulate their host metabolism to gain nutritional resources or may cause host metabolic changes as a consequence of nutrient consumption. Future experiments are necessary to explore to what extent infection-induced shifts to IIS activity serve as an adapted host response to infection versus as a mechanism for pathogens to exploit host physiology for their own gains.

Studies that make use of genetic manipulation have been critical in advancing our understanding of how IIS and innate immune activity interact. While infection studies in *D. melanogaster* IIS mutants have shown varying effects on infection outcome (86, 102, 107), overexpression experiments driving Toll and IMD signaling in the *D. melanogaster* fat body demonstrate an antagonistic response of innate immune signaling on IIS activation (16, 19, 39, 82, 86). Future experiments using tissue-specific or conditionally inducible knockdowns and overexpressions (e.g. 163) will further reveal how IIS affects immune function, including disentangling direct antagonisms from pleiotropic developmental effects and distinguishing tissue autonomy from interorgan communication. Additionally, experiments coupling pharmacological intervention and genetic manipulation of IIS could help elucidate the contributions of TOR- and IIS- mediated effects on immunity. IIS, metabolism, and innate immunity are interrelated in a feedback network, and carefully conceived experiments will be required to disentangle the mechanisms that connect them.

Genetic manipulation of insulin signaling in mosquitos has revealed that activation of IIS can increase production of immune effectors, leading to reduced prevalence of *Plasmodium* infection (117, 118, 122). Overexpression of negative regulators of IIS similarly contributes to reduced *Plasmodium* infectivity (117). It is thus possible that either upregulation or downregulation of IIS could create unfavorable environments for parasite development, increasing the potential for control of disease transmission via genetic manipulation of the mosquito vector. However, direct feeding of insulin to mosquitoes promotes *Plasmodium falciparum* infection success, and the parasite can induce expression of ILPs to suppress the mosquito immune response. The discrepancy between direct feeding of insulin versus genetic manipulation of IIS activity in mosquitos remains to be understood and brings an appropriate note of caution in interpreting individual experiments. Despite these nuances in experimental design, studies that demonstrate how feeding insulin or treating mosquitos with pharmaceutical inhibitors alter metabolic and immune function in mosquitos can inform novel strategies to target for vector pest management.

Common themes of interaction between IIS and immunity have emerged from the body of work described throughout this review. Infection may stimulate or suppress IIS activity to initiate nutrient mobilization in support of an immune response or to preserve metabolic stores. Potentially, we could expect an adapted metabolic response to support varying metabolic needs that could arise when a host is challenged with diverse types of pathogens. Understanding how IIS activity and the innate immune system interact within insects is an exciting avenue of research with compelling foundation but clearly much left to learn.

## Author contributions

AMD: Conceptualization, Investigation, Project administration, Visualization, Writing – original draft, Writing – review & editing. BPL: Investigation, Resources, Supervision, Writing – review & editing.
