# Corrigendum: Interactions between innate immunity and insulin signaling affect resistance to infection in insects

**DOI:** 10.3389/fimmu.2024.1400514

**Published:** 2024-03-21

**Authors:** Andrea M. Darby, Brian P. Lazzaro

**Affiliations:** ^1^Department of Entomology, Cornell University, Ithaca, NY, United States; ^2^Cornell Institute of Host-Microbe Interactions and Disease, Cornell University, Ithaca, NY, United States

**Keywords:** innate immunity, insulin signaling, metabolism, immunometabolism, humoral immunity, Drosophila, mosquito, Bombyx

In the published article, there was an error in the acknowledgments section. The name of Taylar Mouton, one of the people acknowledged for assistance with curating this article, was misspelled as “Taylor” in the published version of the article.

A correction has been made to the Acknowledgments Section. This sentence previously stated:

“Additional thanks to Tory Hendry, Angela Poole, Sri Lakshmi Sravani Devarakonda, Taylor Mouton, and Dorian Jackson for additional comment and feedback on the manuscript.”

The corrected sentence appears below:

“Additional thanks to Tory Hendry, Angela Poole, Sri Lakshmi Sravani Devarakonda, Taylar Mouton, and Dorian Jackson for additional comment and feedback on the manuscript.”

The authors apologize for this error and state that this does not change the scientific conclusions of the article in any way. The original article has been updated.

