# Supplementary material for: Interactions between innate immunity and insulin signaling affect resistance to infection in insects
Source: Front Immunol. 2023 Oct 17;14:1276357. doi: 10.3389/fimmu.2023.1276357 (PMC10616485; doi:10.3389/fimmu.2023.1276357)
Supplement: Supplementary file 2 [file Table_2.docx]

| Tissue | Gene | Genetic manipulation | Effect on insulin activity | Effect on physiology | References |
| --- | --- | --- | --- | --- | --- |
| Fat body | Toll | UAS-Gal4 mediated overexpression | Inhibition of Akt phosphorylation | Reduced levels of triglycerides | Diangelo et al, 2009, Martinez et al, 2020, Roth et al, 2018, Suzawa et al, 2019 |
| Fat body | Relish | UAS-Gal4 mediated overexpression | No effect on Akt phosphorylation | No effect on triglyceride levels | Diangelo et al, 2009 |
| Fat body | Toll | UAS-Gal4 mediated overexpression | Impaired activity of Pdk1 phosphorylation of Akt T342 site | Reduced triglycerides and small body size | Roth et al, 2018 |
| Fat body | Toll | UAS-Gal4 mediated overexpression | Reduced dILP6 expression | Smaller body size as adults, delayed development | Suzawa et al, 2019 |
| Fat body | Toll | UAS-Gal4 mediated overexpression | Upregulation of Kennedy pathway enzymes | Increase in phospholipid synthesis; Expansion of endoplasmic reticulum (ER) and upregulation of ER stress response | Martinez et al, 2020 |
| Fat body | Dif | UAS-Gal4 mediated overexpression | Reduced Akt phosphorylation | Small adult body size | Suzawa et al, 2019 |
| Fat body | Relish | Rel20 mutant and Uas-Gal4 overexpression | Antagonism of FOXO regulation of the lipase brummer | Increased starvation sensitivity | Molaei et al, 2019 |
| Fat body | IMD | UAS-Gal4 mediated overexpression | Reduced Akt phosphorylation | Reduced weight, development, and energetic stores | Davoodi et al, 2019 |
| Ubiquitous | Relish | Rel20 mutant | Altered expression of genes in IIS-dependent metabolism | Increased microbiota loads in gut | Broderick et al, 2014 |
| Ubiquitous | Relish; PGRP-LC; Dredd | Rel20 Mutant; Dredd mutant; PGRP-LC mutant | Reduced Akt phosphorylation, lower expression of *dILP3* | Depletion of fat body lipids, accumulation of midgut lipid droplets | Kamareddine et al, 2018 |
| Enteroendocrine cells in midgut | Relish | UAS-Gal4 RNAi knockdown | Reduced Akt phosphorylation in midgut, *dILP3* transcript reduced | Elevated midgut lipids | Kamareddine et al, 2018 |
| Fat body | Relish | UAS-Gal4 RNAi knockdown | No effect on Akt activity | No effect on lipid content | Kamareddine et al, 2018 |
| Fat body | Relish; Toll | UAS-Gal4 mediated overexpression | Elevated expression of negative IIS regulators *thor* and *Impl2* | Increased lipid droplet size in fat body and mid gut | Harsh et al, 2019 |

**Supplementary Table 2.**

Summary of published studies that test how manipulating innate immune genes affect *Drosophila melanogaster* IIS activity and IIS-dependent metabolism.
